# Hernie médullaire transdurale

**DOI:** 10.11604/pamj.2014.19.375.4041

**Published:** 2014-12-12

**Authors:** Soufiane Belabbes, Mohamed Badaoui, Tarik Salaheddine, Abdennasser El Kharras

**Affiliations:** 1 Service d'Imagerie Médicale, Centre Médico-Chirurgical, Agadir, Maroc; 2 Service de Médecine Interne, Centre Médico-Chirurgical, Agadir, Maroc

**Keywords:** Hernie médullaire transdurale, IRM, myélopathie, Transdurale spinal hernia, MRI, myelopathy

## Abstract

Hernie médullaire transdurale est une case rare de myélopathie. Nous rapportons une observation d'hernie médullaire transdurale chez une femme de 50 ans, tout en insistant sur le rôle de l'IRM dans le diagnostic de cette entité pathologique.

## Introduction

La hernie médullaire transdurale est une cause rare de myélopathie progressivement évolutive. Le diagnostic doit être porté devant une séméiologie typique en IRM médullaire.

## Patient et observation

**Figure 1:**
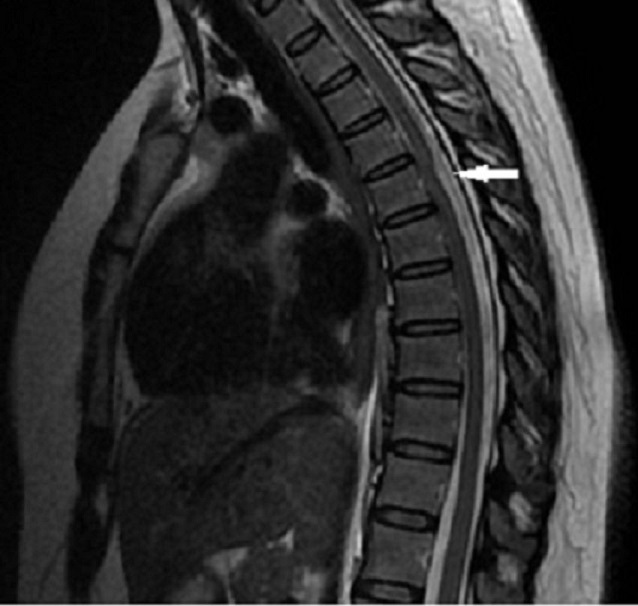
IRM médullaire: coupe sagittale T2 note une déformation médullaire en C avec élargissement de l'espace rétro médullaire, déplacement antérieur du cordon médullaire adhérent au corps vertébral à hauteur de T6 (flèche)

**Figure 2:**
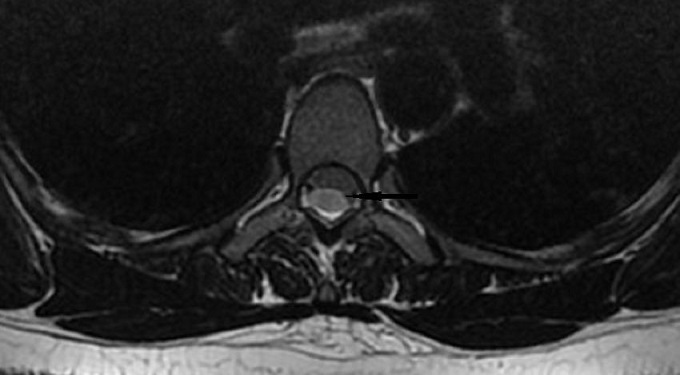
IRM médullaire: coupe axiale T2 passant par T4, montrant le cordon médullaire aspiré vers l'avant et le dehors à gauche, et déformé en livre ouvert ou en éventail (flèche noire). Pas de LCS visible entre le corps vertébral et le cordon médullaire

**Figure 3:**
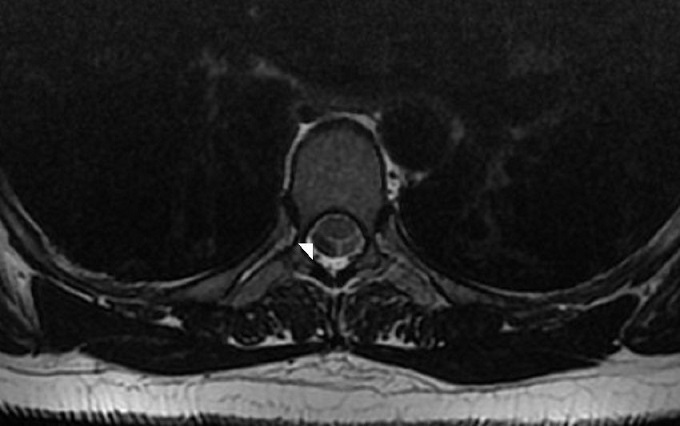
IRM médullaire: aspect en double cordon médullaire (tête de flèche) lié aux artéfacts de flux de LCS éliminant a priori un kyste arachnoïdien

Une femme âgée de 50 ans suivie pour des névralgies cervico-brachiales dans le territoire de C4-C5, s'est présentée à la consultation pour l'apparition d'une monoparésie crurale droite. Un scanner cérébrale a été réalisé et n'a pas montré d'anomalie pouvant expliquer la symptomatologie. L'IRM médullaire a montré à l’étage cervical une uncodiscarthrose modérée aux étages C4-C5 et C5-C6 conflictuelle avec la racine C5 droite. A l’étage dorsal (Figure 1), L'IRM a révélé à hauteur du segment vertébral D4 un déplacement antérieur focal du cordon médullaire, réalisant une déformation en « C », effaçant les espaces sub-arachnoïdiens antérieurs et élargissant les espaces périmédullaire postérieurs, sans signe de souffrance médullaire sous-jacente (Figure 2). L'aspect en double cordon médullaire lié aux artéfacts de flux du liquide cérébro-spinal (LCS) a permis d’éliminer a priori un kyste arachnoïdien et de retenir le diagnostic de la hernie médullaire transdurale (Figure 3). La patiente fut opérée, une plastie dure mérienne a été réalisée permettant de stabiliser la symptomatologie.

## Discussion

La hernie médullaire transdurale (HMT) est une cause rare de myélopathie progressivement évolutive. Cependant depuis l'essor de l'imagerie par résonnance magnétique cette affection est de plus en plus décrite. Le premier cas a été décrit par Wortzman et al en 1974 [1], et depuis plus de 158 cas ont été rapportés dans la littérature [2, 3]. Cette affection est caractérisée par la hernie du cordon médullaire à l′étage thoracique, généralement entre T4 et T7, à travers un défect de la paroi antérolatérale ou antérieure de la dure-mère qui est soit congénital ou acquis.

L′éthiopathogénie des HMT idiopathiques demeure incertaine. Plusieurs phénomènes pourraient être à l'origine d'une brêche durale: les microtraumatismes occultes répétés, le kyste arachnoïdien postérieur congénital, la hernie discale calcifiée érodant la dure-mère, et la duplication dure-mérienne. Une fois la brèche durale s'installe, l′accolement de la moelle à cet orifice est favorisé par sa position anatomique dans le canal rachidien à l’étage thoracique, et est accentué par la pulsatilité physiologique du liquide cérébro-spinal (LCS). Lorsque la moelle est herniée, un phénomène de strangulation est responsable de la symptomatologie clinique.

Un syndrome de Brown-Sequard lentement évolutif est la symptomatologie typique retrouvée dans plus de la moitié des cas [4]. Le premier signe est souvent une monoparésie spastique. D'autres symptômes sont possibles, notamment un syndrome pyramidal unilatéral, des douleurs et des troubles sphinctériens. L'IRM est l'examen de référence dans le diagnostic de la hernie médullaire transdurale. Les séquences sagittales pondérées T2 suffisent le plus souvent pour porter le diagnostic devant la visualisation d'un déplacement antérieur du cordon médullaire, avec une déformation médullaire en C ou en S à l′étage de la hernie, ainsi qu′un élargissement des espaces sous arachnoïdiens postérieurs et un effacement des espaces sous arachnoïdiens antérieurs. Les images sagittales peuvent aussi montrer une atrophie du cordon médullaire associée à un hyper signal témoignant de la souffrance médullaire (myélomalacie), qui engage généralement le pronostic fonctionnel post opératoire. Les coupes axiales montrent la moelle plaquée contre le corps vertébral sans visualisation d′interposition de LCS. Le versant postérieur du cordon médullaire prend un aspect en livre ouvert ou en éventail secondaire au phénomène d′aspiration qui n′est pas présent en cas de kyste arachnoïdien.

Un aspect en “double cordon” médullaire, correspondant à des artefacts de flux de LCS, peut être un indice en faveur d′une hernie transdurale plutôt qu′un kyste arachnoïdien. Le diagnostic de la hernie médullaire a longtemps été source d′erreurs diagnostiques. Mal connu, il était souvent confondu avec son principal diagnostic différentiel, et traité comme tel, le kyste arachnoïdien postérieur. En cas de difficultés diagnostiques, La ciné IRM permet d’écarter le diagnostic de kyste arachnoïdien en montrant la persistance d′un flux de LCS normal à la partie postérieure de la moelle à hauteur de la hernie. Les autres diagnostics différentiels sont: l'atrophie médullaire segmentaire, et l'adhérence médullaire. Le traitement est chirurgical; il permet le plus souvent de stabiliser une symptomatologie évolutive avec même parfois une nette amélioration, notamment si le diagnostic est précoce. La récupération complète est rarement obtenue [5].

Plusieurs techniques sont possibles, la réparation du défect antérieur par plastie dure-mérienne (patch dural) semble être la technique de choix. Parfois un élargissement de l′orifice est réalisé afin d'empêcher une strangulation ultérieure. Lors d'une découverte fortuite chez des patients asymptomatiques, une surveillance clinique rapprochée est indispensable. Un suivi à long terme après la chirurgie est recommandé afin de dépister une éventuelle récidive.

## Conclusion

La hernie médullaire transdurale est une affection rare, souvent méconnue. L'IRM médullaire est l'examen de choix dans le diagnostic de cette pathologie.
